# The First Outbreak Caused by* Acinetobacter baumannii* ST208 and ST195 in China

**DOI:** 10.1155/2016/9254907

**Published:** 2016-04-10

**Authors:** Junyan Qu, Yu Du, Rujia Yu, Xiaoju Lü

**Affiliations:** ^1^Center of Infectious Disease, West China Hospital, Sichuan University, Chengdu 610041, China; ^2^Intensive Care Unit, No. 4 West China Teaching Hospital, Sichuan University, Chengdu 610041, China

## Abstract

This study aimed to analyze the clinical characteristics of patients and molecular mechanisms of the first outbreak mainly caused by sequence types (STs) 208 multidrug resistant (MDR)* Acinetobacter baumannii* in China. A total of 10 clinical samples were collected from 5 patients who were involved in the outbreak. Bacterial identification and antibiotic sensitivity tests were performed by the VITEK-2 COMPACT automated system. MICs of tigecycline for clinical isolates were determined using broth microdilution. The clonal relatedness of* A. baumannii* clinical isolates in our local settings was determinated by pulsed-field gel electrophoresis (PFGE) and multilocus sequence typing (MLST). A total of 7* A. baumannii* strains were isolated and all were MDR strains; two of them were carbapenem-nonsusceptible strains.* bla*_*OXA-23*_ was the only acquired carbapenemase gene in the isolates. The isolates belonged to a single clonal pulsotype determined by PFGE and two sequences types (STs) determined by MLST. The isolates belonged to the globally disseminated clonal complex 92, among which ST195 and ST208 were the most common sequence types (71.43% and 28.57%). The outbreak was successfully controlled by stringent infection control measures, especially improving the hand hygiene compliance and enhancing antimicrobial stewardship. In conclusion, this is the first description of an outbreak caused mainly by* A. baumannii* of ST208 in China. Infection control measures should be strengthened when infection outbreaks in hospital.

## 1. Introduction

*Acinetobacter baumannii (A. baumannii)* is an important opportunistic nosocomial pathogen, especially in patients in intensive care unit (ICU) [1]. It can cause various infections such as pneumonia, endocarditis, and skin and wound infections [2]. There are many risk factors for* A. baumannii* infections such as trauma, mechanical ventilation, immunosuppression, long-term hospitalization, invasive interventions, and the use of antimicrobial drugs [2, 3]. The efficacy of carbapenems against multidrug resistant (MDR)* A. baumannii* has been undermined by the emergence of carbapenemase-producing strains [4]. The prevalence of MDR* A. baumannii* has been increasing worldwide, which poses a serious challenge for infection control and clinical management [5, 6].

Outbreaks of MDR* A. baumannii* including OXA-23 carbapenemase-producing* A. baumannii* have been reported worldwide [7–11]. The most widely distributed clonal complex (CC) of carbapenem-resistant* A. baumannii* was CC92 in most Asian locales including China [8, 12]. ST208 belonging to the CC92 had not been identified in China before 2011, but recently it had been found successively in Guangdong, Sichuan, and Zhejiang provinces of China and has become one of predominant sequence types (STs) [9, 13, 14].

The aim of this study was to analyze the clinical characteristics of patients and molecular mechanisms of the first outbreak mainly caused by ST208 multidrug resistant OXA-23 carbapenemase-producing* Acinetobacter baumannii* observed among patients in western China.

## 2. Materials and Methods

### 2.1. Clinical Setting and Patients

The No. 4 West China Teaching Hospital, Sichuan University, is a 400-bed university hospital with a 7-bed ICU, which is specialized for occupational diseases. The working situation is characterized by an average of 8 nurses, backed up by 5 intensive care physicians in the ICU. The ICU has one common room with two sinks. The common room can be separated into six private isolated spaces by curtains. The admittance visitors are limited. This outbreak involved five cases. They were all in critical illness with endotracheal intubation and endotracheal aspiration. Their lower respiratory tract secretions were collected. Patients' clinical data were recorded.

### 2.2. Bacterial Strains

On April 23, 2013, 5 MDR* A. baumannii* strains were isolated from 5 patients from the ICU of this hospital. On May 4, 2013, 5 clinical samples were collected from the same five patients, and 2 MDR* A. baumannii* strains were isolated from 2 patients, respectively. A total of 7 clinical isolates were recovered.

### 2.3. Bacterial Identification and Antimicrobial Susceptibility Testing

Bacterial identification was performed by the VITEK-2 COMPACT automated microbiology system (BioMérieux). Species identifications were established by detecting and sequencing* recA* and* gyrB* genes. Minimal inhibitory concentrations (MICs) of meropenem, imipenem, ceftazidime, cefepime, piperacillin, amoxicillin/clavulanate, aztreonam, colistin, amikacin, ciprofloxacin cotrimoxazole, gentamicin, and nitrofurantoin against* A. baumannii* isolates were also tested by the VITEK-2 COMPACT automated microbiology system, and the results were interpreted according to Clinical and Laboratory Standards Institute (CLSI) guidelines [15]. The MIC of each isolate to tigecycline was determined using broth microdilution method. The FDA approved tigecycline breakpoints for the Enterobacteriaceae (susceptible ≤ 2 mg/L; intermediate = 4 mg/L; resistant ≥ 8 mg/L) were used as provisional MIC breakpoints in this study [16].* Escherichia coli* ATCC25922 was used as quality control for susceptibility testing. Susceptibility testing for each isolate was performed in triplicate.

### 2.4. PCR Amplifications and Sequence Analysis of Carbapenemase-Encoding Genes

Detection of classes A and D carbapenemase genes (*bla*_*GES*_,* bla*_*KPC*_,* bla*_*OXA-51*_,* bla*_*OXA-23*_,* bla*_*OXA-24*_,* bla*_*OXA-58*_, and* bla*_*OXA-143*_) and detection of class B metallo-*β*-lactamases genes (*bla*_*IMP*_ *, bla*_*VIM*_ *, bla*_*SPM*_ *, bla*_*GIM*_ *, bla*_*SIM*_, and* bla*_*NDM*_) were performed by polymerase chain reaction (PCR) method as previously described [13, 17–21]. Purified PCR products were sequenced in both directions with the ABI 3730 automated sequencer (Applied Biosystems, Warrington, United Kingdom). The nucleotide sequences were analyzed using BLAST programs at the NCBI website (http://www.ncbi.nlm.nih.gov/).

### 2.5. Pulsed-Field Gel Electrophoresis (PFGE) and Multilocus Sequence Typing (MLST)

PFGE was employed to determine clonal relatedness of the isolates and was performed with ApaI-digested genomic DNAs using the DRII PFGE system (Bio-Rad, Marnes-la-Coquette, France), as previously described [22], with minor modifications. The conditions of electrophoresis were as follows: temperature of 14°C, voltage of 6 V/cm, run time of 20 h, and switch time of 5 to 35 s. PFGE results were analyzed by eye according to the criteria of Tenover et al. [23]. Strains were considered closely related and of the same pulsotype if their restrictive enzymatic maps are three or fewer band shifts. MLST was performed to identify the sequence types (STs) of the isolates. Seven housekeeping genes,* gltA*,* gyrB*,* gdhB*,* recA*,* cpn60*,* gpi*, and* rpoD*, were amplified and sequenced as described previously [13, 24]. The sequence of each allele was compared in PubMLST database (http://pubmlst.org/abaumannii/) and STs were designated according to the allelic profiles. The relatedness among the different STs was assessed by the use of the eBURST program (version 3, http://eburst.mlst.net/).

## 3. Results

### 3.1. Outbreak Description

In April 2013 in a county in western China, 36 workers suffered from ammonia poisoning during an ammonia leak accident. Five of them were initially hospitalized in an ICU of a local hospital; after a day or two, they were referred to the ICU of a university hospital in western China successively. Their main clinical symptoms were chest congestion, cough, pink frothy sputum after inhaling ammonia immediately, and then a coma. Endotracheal intubation was done in local hospital. On physical examination, diffuse moist crackles and rhonchi were heard in both lungs in all patients. Each of the 5 patients had pulmonary infection and chemical pneumonitis according to medical history and laboratory, radiologic, and clinical findings. Cephalosporins or respiratory fluoroquinolones were used for the empirical treatment of pulmonary infection in local hospital. The characteristics of the five patients involved in the outbreak were shown in Table 1. Two patients finally died of multiple organ failure. One of the two patients had cardiopulmonary arrest on admission. Another patient died 118 days after being hospitalized in ICU.

Within 24 hours of admission to the university hospital, the 5 clinical specimens were first collected. However, all the 5 patients had a history of previous hospitalization in other hospitals; 48 hours later, the first collection of five specimens was obtained. Therefore, the resistant strains were characterized as hospital acquired. The index case of this outbreak was unclear. It was possible that this pathogen has been introduced into the ICU environment of local hospital by a patient, and then it might be transmitted to the 5 patients through medical workers' hands, or one of the 5 patients was index case and other patients were infected thereafter. Stringent infection control measures were implemented including audit of hand hygiene compliance, access restrictions to affected areas, the use of disposable gloves, disinfection of surroundings, self-protection for healthcare workers, and waste management. Enhanced antimicrobial stewardship was introduced and antimicrobial agents were adjusted with the guidance of experts in infectious diseases.

Until now, carbapenemase-producing MDR* Acinetobacter baumannii* have not been isolated from other cases in this unit and the outbreak was successfully controlled.

### 3.2. Susceptibility Testing

The results of 14 antimicrobic agents susceptibility test in 7 clinical isolates of* Acinetobacter baumannii* were shown in Table 2. All* A. baumannii* were MDR strains and resistant to piperacillin, ceftazidime, ciprofloxacin, gentamicin, and cotrimoxazole but sensitive to colistin and tigecycline. Of note, all isolates were resistant to carbapenems except isolates A1 and A2.

### 3.3. Carbapenemase-Encoding Genes, PFGE, and MLST

PCR and sequencing confirmed that* bla*_*OXA-23*_ was the only acquired carbapenemase gene in all isolates. PFGE clustered the OXA-23-producing* A. baumannii* isolates into a single PFGE clonal type. Two STs were identified by MLST among the 7* A. baumannii* isolates. Interestingly, the sequence type of the first isolated 5 clinical strains (A1, A3, A4, A6, and A7) was ST208 (1-3-3-2-2-97-3); however, the sequence type of the second isolated 2 clinical strains (A2 and A5) was ST195 (1-3-3-2-2-96-3), and only the 305-bp* gpi* locus of* A. baumannii* changed from the allele 97 to 96. According to MLST eBURST analysis, ST208 and ST195 isolates involved in the outbreak carrying* bla*_*OXA-23*_ are considered to belong to the globally disseminated clonal complex (CC) 92.

## 4. Discussion

To our knowledge, we describe the first outbreak in western China that was mainly caused by* Acinetobacter baumannii* of ST208 carrying the OXA-23 carbapenemase. Outbreaks of OXA-23 carbapenemase-producing* A. baumannii* have been reported all over the world [9, 13, 14]. ST208 had been identified in the United States, Japan, China, and Denmark through molecular epidemiological studies [12–14, 25–27]; however, outbreak that was caused by* A. baumannii* of ST208 has not been reported. All our ST208 isolates carried* bla*_*OXA-23*_. Although* A. baumannii* of ST208 could only be detected in recent years, ST208 is becoming one of predominant STs of carbapenem-nonsusceptible isolates in the United States and China [12–14, 26]. Whether* A. baumannii* of ST208 has more virulence than other ST type strains was unclear; however, we should raise awareness to prevent spread if they were detected.* bla*_*OXA-23*_ was detectable in every isolate in this study including carbapenem-susceptible isolate and it was the only acquired carbapenemase gene in all isolates. Previous studies have shown that* bla*_*OXA-23*_ was the most common carbapenemase gene in China [28] and it was also detectable in carbapenem-susceptible isolates [29]. Though acquisition of OXA carbapenemases was one of the predominant mechanisms for carbapenem resistance in* Acinetobacter* spp., however, their expression was regulated by upstream promoters such as insertion sequence (IS) IS*Aba1* [30, 31]. So there were some isolates carrying OXA-23 carbapenems that were sensitive to carbapenem.

It was the first time to identify* A. baumannii* of ST195 in western China. Interestingly,* A. baumannii* of ST208 and ST195 were isolated successively from the same patients with difference only in the* gpi* locus. In addition, the* gpi* locus of* A. baumannii* appeared to be diverse compared to other loci according to the* Acinetobacter* MLST database (http://pubmlst.org/abaumannii/). Therefore,* gpi* might not be an ideal locus for typing which was similar to the finding of the previous studies [13, 32].

This outbreak occurred in a public unit. The five patients involved in this outbreak had risk factors for infection with MDR* Acinetobacter* species, such as underlying severity of illness, exposure to ICU, invasive procedures, and receipt of mechanical ventilation [6]. They stayed in different hospitals in a short term; we did not know whether the surface cleaning equipment (detergent solution, cleaning cloths, and mop) and the sanitary equipment contaminated MDR strains, but MDR strains contamination of the surface cleaning equipment was more frequent than contamination of the sanitary equipment. On treatment outcome, one patient improved, perhaps because she had less risk factors than others and she was infected with carbapenem-susceptible* A. baumannii*. Two of the five patients finally died. Their poor clinical outcomes might be, in part, due to severe trauma, hypoimmunity, longer stay in hospital ICU, coinfection of other bacteria in the course of the disease, and so on [6]. These MDR strains of* A. baumannii* encountered in this outbreak were resistant to most antibiotics. Though they were sensitive to tigecycline* in vitro*, maybe the emergence of resistance during treatment influenced the therapy results [33]. Colistin can increase the cure rate and improve the prognosis of severely ill patients with MDR* Acinetobacter* species infections [34]; maybe early initiation of colistin therapy was a good option, but we could not get it at that time in China.

We successfully controlled the outbreak. Infection control measures in this outbreak were strengthened; for instance, we improved the hand hygiene compliance of medical personnel by multimodal strategies including administrative support, motivation, free availability of hand disinfectants, training, and intensive education of medical workers. Many evidences suggested that improving the hand hygiene compliance could significantly reduce the rates of hospital acquired infections in a hospital [35]. Furthermore, antimicrobial stewardship, including the choice of antibiotic and dosage regimen, was another important measure, which could also reduce the development of antibiotic resistance in critical care units [36].

The presence of* bla*_*OXA-23*_ genes in carbapenem-susceptible* A. baumannii* highlights the threat of infection control, because some carbapenemase-encoding genes might not be detected and these susceptible organisms were likely to be ignored. In addition,* A. baumannii* strains could carry the* bla*_*OXA-23-like*_ genes on plasmids and transferred horizontally to other species. Therefore, infection control measures including rapid identification of* bla*_*OXA-23*_, hand hygiene, and environmental disinfection should be reinforced to reduce the further spread of* A. baumannii*.

## 5. Conclusions

We first reported an outbreak of infection mainly caused by ST208 OXA-23 carbapenemase-producing* Acinetobacter baumannii* in the ICU of a hospital in western China. ST208 and ST195* A. baumannii* were isolated successively from the same patients and the diversity of* A. baumannii* suggested that the* gpi* gene as one of* A. baumannii* MLST might need to be reconsidered. Infection control measures especially hand hygiene and antimicrobial stewardship should be strengthened when OXA carbapenemase-producing* A. baumannii* including carbapenem-susceptible strains were isolated in a hospital.

## Figures and Tables

**Table 1:** Characteristics of the five patients infected with multidrug resistant (MDR) *Acinetobacter baumannii* strains.

Patient	Isolate number	Age (years)/sex	Type of specimen	Date of isolation	Underlying disease/predisposing	Length of hospitalization in unit (days)	Laboratory findings	Radiologic finding	Type of infection	Antimicrobial used as treatment for infection	outcome
1	A1	40/F	Respiratory tract secretions	2013/04/22	AAT, CP, EI, T, and PN	57	Elevation of PCT, CRP, and leukocytosis; positive culture (tracheal aspirate)	Diffuse bilateral exudation with patchy and mass	Pneumonia	SCF/MEM	Improved
	A2		Respiratory tract secretions	2013/05/05	AAT, CP, EI, T, and PN	—	Elevation of PCT, CRP, and leukocytosis; positive culture (tracheal aspirate)	Diffuse bilateral exudation with patchy and mass	Pneumonia	MEM	—

2	A3	49/F	Respiratory tract secretions	2013/04/22	AAT, CP, EI, T, PN, and AAB	118	Elevation of PCT, CRP, and leukocytosis; positive culture (tracheal aspirate)	Diffuse bilateral exudation with patchy and mass; pneumomediastinum	Pneumonia	SCF/TGC/AK	Deceased

3	A4	42/F	Respiratory tract secretions	2013/04/22	AAT, CP, EI, T, PN, CRP, and AAB	52	Elevation of PCT, CRP, and leukocytosis; positive culture (tracheal aspirate)	Diffuse bilateral exudation with patchy and mass	Pneumonia	SCF/TGC/AK	Deceased

4	A5	41/F	Respiratory tract secretions	2013/04/22	AAT, CP, EI, T, and PN	59	Elevation of PCT, CRP, and leukocytosis; positive culture (tracheal aspirate)	Diffuse bilateral exudation with patchy and mass	Pneumonia	SCF/AK	Discharged
	A6		Respiratory tract secretions	2013/05/05	AAT, CP, EI, T, PN, and CVC	—	Elevation of PCT, CRP, and leukocytosis; positive culture (tracheal aspirate)	Diffuse bilateral exudation with patchy and mass	Pneumonia	TGC	—

5	A7	42/F	Respiratory tract secretions	2013/04/22	AAT, CP, EI, T, and PN	104	Elevation of PCT, CRP, and leukocytosis; positive culture (tracheal aspirate)	Diffuse bilateral exudation with patchy and mass	Pneumonia	SCF/TGC/TZP	Discharged

F, female; AAT, acute ammonia intoxication; CP, chemical pneumonitis; EI, endotracheal intubation; T, tracheotomy; PN, parenteral nutrition; AAB, anhydrous ammonia burns; CPR, after cardiopulmonary resuscitation; CVC, central venous catheters; PCT, procalcitonin; CRP, C-reactive protein; TGC, tigecycline; SCF, cefoperazone sulbactam; AK, amikacin; MEM, meropenem; TZP, piperacillin-tazobactam.

**Table 2:** Antimicrobial susceptibility of multidrug resistant (MDR) *A. baumannii* strains from a hospital in western China.

Antibiotic	MIC (mg/L)				
	A1 and A2	A3	A4	A5 and A6	A7
Imipenem	2	≥16	≥16	≥16	≥16
Meropenem	2–4	≥16	≥16	≥16	≥16
Cefepime	8–64	≥64	≥64	≥64	≥64
Ceftazidime	≥64	≥64	≥64	≥64	≥64
Piperacillin	≥128	≥128	≥128	≥128	64
Amoxicillin/clavulanate	≥32	≥32	≥32	≥32	≥32
Tigecycline	1-2	2	2	0.5–2	0.25
Colistin	0.5–1	1	1	0.5–1	0.5
Amikacin	≥64	≥64	64	64	≥64
Ciprofloxacin	≥4	≥4	≥4	≥4	≥4
Cotrimoxazole	≥320	≥320	≥320	≥320	≥320
Gentamicin	≥16	≥16	≥16	≥16	≥16
